# Danhong Injection Alleviates Cardiac Fibrosis via Preventing the Hypermethylation of Rasal1 and Rassf1 in TAC Mice

**DOI:** 10.1155/2020/3158108

**Published:** 2020-12-29

**Authors:** Sinai Li, Ping Li, Weihong Liu, Juju Shang, Shenglei Qiu, Xiang Li, Wei Liu, Haoyue Shi, Mingxue Zhou, Hongxu Liu

**Affiliations:** ^1^Beijing Hospital of Traditional Chinese Medicine, Capital Medical University, Beijing 100010, China; ^2^Beijing Institute of Traditional Chinese Medicine, Beijing 100010, China

## Abstract

**Background/Aim:**

Danhong injection (DHI) is a Chinese patent drug used for relieving cardiovascular diseases. Recent studies have suggested that DNA methylation plays a pivotal role in the maintenance of cardiac fibrosis (CF) in cardiovascular diseases. This study was aimed at identifying the effect and the underlying mechanism of DHI on CF, especially the DNA methylation.

**Methods:**

A CF murine model was established by thoracic aortic constriction (TAC). A 28-day daily treatment with or without DHI via intraperitoneal injection was carried out immediately following TAC surgery. The changes in cardiac function, pathology, and fibrosis following TAC were measured by echocardiography and immunostaining. We used methyl-seq analysis to assess the DNA methylation changes in whole genes and identified the methylation changes of two Ras signaling-related genes in TAC mice, including Ras protein activator like-1 (Rasal1) and Ras-association domain family 1 (Rassf1). Next, the methylation status and expression levels of Rasal1 and Rassf1 genes were consolidated by bisulfite sequencing, quantitative reverse transcription polymerase chain reaction (RT-qPCR), and Western blotting, respectively. To determine the underlying molecular mechanism, the expressions of DNA methyltransferases (DNMTs), Tet methylcytosine dioxygenase 3 (TET3), fibrosis-related genes, and the activity of Ras/ERK were measured by RT-qPCR and Western blotting.

**Results:**

DHI treatment alleviated CF and significantly improved cardiac function on day 28 of TAC. The methyl-seq analysis identified 42,606 differential methylated sites (DMSs), including 19,618 hypermethylated DMSs and 22,988 hypomethylated DMSs between TAC and sham-operated mice. The enrichment analysis of these DMSs suggested that the methylated regulation of Ras signal transduction and focal adhesion-related genes would be involved in the TAC-induced CF development. The results of bisulfite sequencing revealed that the TAC-induced methylation affected the CpG site in both of Rasal1 and Rassf1 genes, and DHI treatment remarkably downregulated the promoter methylation of Rasal1 and Rassf1 in CF hearts. Furthermore, DHI treatment upregulated the expressions of Rasal1 and Rassf1, inhibited the hyperactivity of Ras/ERK, and decreased the expressions of fibrosis-related genes. Notably, we found that DHI treatment markedly downregulated the expression of DNMT3B in CF hearts, while it did not affect the expressions of DNMT1, DNMT3A, and TET3.

**Conclusion:**

Aberrant DNA methylation of Rasal1 and Rassf1 genes was involved in the CF development. DHI treatment alleviated CF, prevented the hypermethylation of Rasal1 and Rassf1, and downregulated DNMT3B expression in CF hearts.

## 1. Introduction

Cardiac fibrosis (CF) is an integral constituent of every form of chronic heart disease, and its relevance for disease progression is increasingly being recognized [1]. CF is defined as excess deposition of the extracellular matrix (ECM), resulting in tissue scarring and organ dysfunction. In recent years, although the underlying mechanisms of CF are still unknown, numerous studies suggested a pivotal role of DNA methylation in CF [2]. Ras protein activator like-1 (RASAL1) and Ras-association domain family 1 (RASSF1) function as inhibitors of Ras signaling, and the aberrant promoter methylation of RASAL1 and RASSF1 contributes to pressure overload-induced CF [3, 4]. Xu et al. found that increased fibrosis was associated with significantly elevated promoter methylation of RASAL1, decreased RASAL1 expression, and increased Ras-GTP activity [3]. Tao et al. reported that upregulation of p-ERK1/2 was detected in activated cardiac fibroblasts with decreased RASSF1A expression, which is important for pathogenesis of CF and activation of fibroblasts [4]. Moreover, administration of the DNA methylation inhibitor could relieve the CF and hypertrophy and improve the cardiac function [5]. However, a highly efficient, specific, and safe compound targeting the aberrant DNA methylation in CF patients has not been identified yet [6].

Danhong injection (DHI), a Chinese herbal medicine composed of *Salvia miltiorrhiza* and *Carthamus tinctorius*, has been industrialized, patented, and well characterized by a dual-standard quality assessment [7]. *Salvia miltiorrhiza* and *Carthamus tinctorius* have been used widely and successfully to treat patients with cardiovascular disease in China [8]. A systematic review and meta-analysis comprising 13 randomized controlled trails (RCTs) revealed that DHI significantly reduced the risk of mortality, recurrent angina, arrhythmia, and heart failure, accompanied by improved cardiac function in patients with cardiovascular disease [9]. The methylation regulatory effect of DHI has been reported in a postmyocardial infarction (MI) mouse model [10]. Salvianolic acid B, a main component of DHI, was reported to inhibit liver fibrosis by epigenetically regulating Patched1 and downregulating DNMT1 [11]. These studies demonstrated that epigenetical inhibition of DNA methylation may be the target of antifibrosis effect of DHI. However, the effect and mechanism of DHI on CF in thoracic aortic constriction (TAC) mice have not been fully investigated. Therefore, we firstly used methyl-seq analysis to overview the DNA methylation changes in whole genes and identified the aberrant promoter methylation of two Ras signaling-related genes in TAC mice, Rasal1 and Rassf1, which were reported as a significant determinant of CF [3, 12]. Based on the results, we further identified the antifibrosis effect of DHI and the underlying mechanism in hypermethylation of Rasal1 and Rassf1 in TAC mice.

## 2. Materials and Methods

### 2.1. Drug Preparation and Quality Control

DHI (batch number: 17041018) was provided by Heze Buchang Pharmaceutical Co. Ltd. (Shaanxi, China). DHI was approved by the Chinese Food and Drug Administration (CFDA) as a Chinese herbal patented product for coronary heart disease patients and listed in the Chinese Pharmacopoeia (approval no. Z20026866). The ultraperformance liquid chromatography-time-of-flight mass spectrometry (UPLC-TOF-MS) was employed to quantify 7 representative constituents of DHI, including salvianolic acid A (PubChem CID: 5281793), salvianolic acid B (PubChem CID: 6451084), salvianolic acid D (PubChem CID: 75412558), kaempferide (PubChem CID: 5281666), coumalic acid (PubChem CID: 68141), rosmarinic acid (PubChem CID: 5281792), and protocatechualdehyde (PubChem CID: 8768). Total ion chromatograms of DHI based on UPLC-TOF-MS are shown in Supplementary Figure 1.

### 2.2. Animals

Male C57BL/6N mice, 6-8 weeks old, were purchased from Beijing Vital River Laboratory Animal Technology Co., Ltd. (Beijing, China). The mice were housed in cages at 22 ± 2°C, with humidity of 40 ± 5% and under a 12 h light/dark cycle with standard diet and water ad libitum. The present study was conducted in accordance with the Guide for the Care and Use of Laboratory Animals published by the National Institutes of Health (NIH, Bethesda, MD, USA). The experiment protocols were approved by the Institutional Animal Care and Use Committee (IACUC) of Beijing Hospital of Traditional Chinese Medicine (Beijing, China).

### 2.3. Establishment of a CF Murine Model

A CF murine model was established via TAC as previously described [13]. Briefly, the mice were anesthetized with isoflurane after fasting for 12 h. The mice were then orally intubated with 20-gauge tubing and ventilated (Kent Scientific Corp., Torrington, CT, USA) at 120 breaths/min with 0.1 mL of tidal volume. A 3 mm left-side thoracotomy was created at the second intercostal space. The transverse aortic arch was ligated (7-0 Prolene) between the innominate and left common carotid arteries with an overlying 27-gauge needle, and then, the needle was removed, and a discrete region of stenosis was left. The chest was closed, and the left-side pneumothorax was evacuated. Perioperative (24 h) mortality was less than 10%. The animals in the sham-operated group underwent the same procedure, but without constriction.

### 2.4. Drug Administration

After TAC surgery, mice were randomly assigned into four groups: sham-operated (sham, *n* = 8), TAC-operated (TAC, *n* = 8), low-dose DHI (L-DHI, *n* = 8), and high-dose DHI (H-DHI, *n* = 8). Two doses of DHI comprising 6.0 (L-DHI) and 12.0 (H-DHI) mL/kg were selected. After TAC surgery, mice were daily treated with DHI intraperitoneally (i.p.) for 28 days. Besides, the negative control group received normal saline (12 mL/kg).

### 2.5. Echocardiography

Echocardiography was performed at 28 days after TAC using a Vevo 770 ultrasound system (VisualSonics, Toronto, Canada) equipped with a real-time microvisualization scan head probe (RMV-707B) that operated at a central frequency of 30 MHz. Mice were anesthetized with isoflurane, at a concentration of 4% (induction) and 1.5% (maintenance) in 100% oxygen. Each animal was placed on a heating table (32°C) in a supine position with the extremities tied to the table through a 4-lead electrocardiogram. The heart rate (HR) was recorded simultaneously. Moreover, the parasternal short-axis view was obtained at the level of papillary muscles to measure left ventricle (LV) endocardial and epicardial areas in diastole and systole. These measurements were obtained by tracing the endocardial and epicardial border of the LV, where the papillary muscles were excluded from the endocardial tracings. LV internal dimensions at diastole/systole (LVIDd/LVIDs) and LV anterior/posterior wall thickness (LVAW/LVPW) were measured and used to calculate ejection fraction (EF) and fractional shortening (FS). The peak velocity of the septal basal level and the posterior wall were measured using tissue Doppler from a four-chamber and short-axis view, respectively. Early diastolic mitral annular velocity (Ea) and late diastolic mitral annular velocity (Aa) were measured, and the ratio of Ea/Aa was calculated. Measurements were performed in accordance with the guidelines presented by the American Society of Echocardiography and were acquired at HR of 400-450 beats per minute (bpm). At least three measurements were carried out, and the values were averaged for each parameter.

### 2.6. Histopathology

Cardiac specimens were fixed in 10% neutral buffered formalin and then embedded into paraffin for light microscopic examination and sectioned at a thickness of 8 *μ*m. Hematoxylin and eosin (H&E) staining (Abcam, Cambridge, UK), Masson's trichrome staining (Sigma-Aldrich, St. Louis, MO, USA), and Picrosirius red staining (Abcam, Cambridge, UK) kits were used to examine the changes in cardiac pathology and the collagen deposition in myocardial tissue. Each section was assessed under light microscopic fields, and images were taken using an inverted microscope (Zeiss, Oberkochen, Germany). The fibrotic areas were measured 28 days after TAC surgery on Masson's trichrome-stained slides.

### 2.7. Western Blotting

Cardiac tissues were lysed with lysis buffer (Beyotime Institute of Biotechnology, Shanghai, China). Total protein (30 *μ*g) from samples was then fractionated by electrophoresis through 10% sodium dodecyl sulfate-polyacrylamide gel electrophoresis (SDS-PAGE). Proteins were run at 120 V for 1.5 h before transferring onto polyvinylidene difluoride (PVDF) membranes. After blocking with 5% BSA, the membranes were incubated with DNMT1, DNMT3A, DNMT3B, TET3, RASSF1, RASAL1, *α*-smooth muscle actin (*α*-SMA), transforming growth factor-beta1 (TGF-*β*1), total ERK1/2, p-ERK1/2, and glyceraldehyde 3-phosphate dehydrogenase (GAPDH) primary antibodies. Following incubation with primary antibodies, blots were washed thrice with TBS/Tween-20 before incubation for 1 h in goat anti-mouse or goat anti-rabbit horseradish peroxidase- (HRP-) conjugated antibody at 1 : 5000 dilution in TBS/Tween-20 containing 5% skimmed milk. After extensive washing in TBS/Tween-20, the blots were processed with distilled water for detection of antigen, and densitometric analysis was carried out using image acquisition and analysis software (Tanon, China).

### 2.8. Quantitative Reverse Transcription Polymerase Chain Reaction (RT-qPCR)

RNA was extracted from heart tissue using an ultrapure RNA kit (CWBio, Beijing, China) and then reversely transcribed with a HiFi-MMLV cDNA kit (CWBio, Beijing, China). The RT-qPCR was undertaken using a SYBR Green PCR Master Mix kit (CWBio, Beijing, China), with GAPDH as the housekeeping gene. Fold changes in the mRNA levels were calculated using the 2^-*ΔΔ*CT^ method. All assays were performed according to the manufacturer's instructions. The sequences of the primers used are listed in Table 1.

### 2.9. Methyl-seq Data Analysis

Total DNA was extracted from the mouse heart from TAC (*n* = 4) and sham-operated (*n* = 4) groups using the AllPrep DNA/RNA Mini Kit (Qiagen). The DNA samples were processed using an Agilent SureSelect Methyl-seq Target Enrichment System (Agilent Technologies Inc., Santa Clara, CA, USA) to enrich the targets of interest. Briefly, 3 *μ*g of DNA calculated by Qubit was fragmented by sonication. Subsequently, library preparation was performed with the SureSelect^XT^ Methyl-Seq Reagent Kit (Agilent Technologies Inc., Santa Clara, CA, USA). DNA was bisulfite-treated using the EZ DNA Methylation-Gold Kit (Zymo Research, Irvine, CA, USA). Sequenced libraries were assessed by Bioanalyzer and quantified with the KAPA Library Quantification Kit (Roche, Basel, Switzerland). The libraries were then sequenced on the HiSeq X Ten System (Illumina Inc., San Diego, CA, USA) with 150 bp pair-end reads, generating 120-172 million reads per sample.

General quality control checks were performed with FastQC v0.8.0 (http://www.bioinformatics.babraham.ac.uk/projects/fastqc/). Each dataset was filtered for average base quality score (>20). Filtered datasets were aligned to a reference genome using Bismark 0.7.8 software [14]. The reference genome index contained the genome sequence of enterobacteria phage *λ* (NC_001416.1) in addition to all chromosomes of the mm10 assembly. Mappings for all datasets generated from the same library were merged, and duplicates removed via the Bismark deduplication tool (deduplicate_bismark_alignment_output.pl). Mapped reads were then separated by genome (phage *λ*) and by source strand (plus or minus). Finally, SAM alignments for multiple libraries from the same animal were merged. Read pairs mapped to phage *λ* were used to confirm that the observed bisulfite conversion rate was >99%. Read pairs mapped to the mm10 reference genome were used for downstream analysis.

Using the DSS R package (v2.15.0) [15], differential methylated sites (DMSs) were identified by DSS with the call DML function (default parameters). DMS calls were made via metilene [16], with a *P* value threshold of 0.05 and mean methylation difference of 10%. The genes annotated by DMSs were imported into the clusterProfiler package [17] for Gene Ontology (GO) and Kyoto Encyclopedia of Genes and Genomes (KEGG) pathway enrichment analyses and data visualization. To assess the interactions between DMSs, the protein-protein interactions (PPI) between DMSs were extracted from the STRING V10 database [18].

### 2.10. Bisulfite Sequencing PCR

200 ng of DNA was bisulfite-treated using the EZ DNA Methylation-Gold Kit (Zymo Research) according to the supplier's instruction. PCR was performed with LA Taq HS (Takara). PCR products were cloned into a pCR4-TOPO vector (Invitrogen). After transformation, cells were spread on ampicillin-containing agar plates and 10-20 colonies were chosen for analysis. Analysis of DNA methylation statues was carried out using the web-based software QUMA [19]. The bisulfite sequencing PCR primers are listed in Table 2.

### 2.11. Statistical Analysis

All data were expressed as mean ± standard error of the mean (SEM). The two groups were compared using Student's *t*-test. For the statistical analysis of data from multiple groups, one-way analysis of variance (ANOVA) was used followed by the Bonferroni post hoc test. For data that did not meet Student's *t*-test or ANOVA assumptions, the Kruskal-Wallis nonparametric test was applied. The GraphPad Prism 6.0 (GraphPad Software Inc., San Diego, CA, USA) and SPSS 16.0 (IBM, Armonk, NY, USA) software was used for data analysis. *P* < 0.05 was considered statistically significant.

## 3. Results

### 3.1. DHI Treatment Ameliorated Cardiac Function and Alleviated Cardiac Hypertrophy in TAC Mice

**Figure 1:**
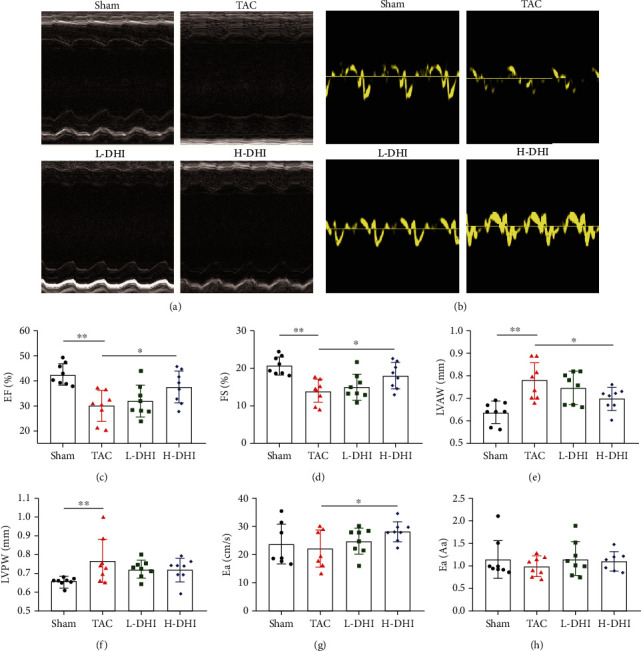
DHI treatment improved cardiac function and alleviated cardiac hypertrophy in TAC mice. (a) Representative LV M-mode tracing images of hearts from sham, TAC, L-DHI, and H-DHI mice. (b) Representative LV tissue Doppler images of hearts from sham-operated, TAC, L-DHI, and H-DHI mice. (c) EF was compared among groups on day 28 after TAC (*n* = 8). (d) FS was compared among groups on day 28 after TAC (*n* = 8). (e) LVAW was compared among groups on day 28 after TAC (*n* = 8). (f) LVPW was compared among groups on day 28 after TAC (*n* = 8). (g) Ea was compared among groups on day 28 after TAC (*n* = 8). (h) Ea/Aa was compared among groups on day 28 after TAC (*n* = 8). ^∗^*P* < 0.05. ^∗∗^*P* < 0.01.

The echocardiography was undertaken at day 28 after TAC to evaluate the effect of DHI on cardiac function (Figures 1(a) and 1(b)). The LV-EF (*P* < 0.01) and LV-FS (*P* < 0.01) in the TAC group were significantly declined compared with those in the sham-operated group, which suggested that the TAC surgery impaired the cardiac systolic function. Besides, the LVAW (*P* < 0.01) and LVPW (*P* < 0.01) in the TAC group were remarkably increased compared with those in the sham-operated group, indicating that cardiac hypertrophy was induced by TAC. After DHI treatment, the LV-EF (*P* < 0.05) and LV-FS (*P* < 0.05) in the H-DHI group were both improved compared with those in the TAC group, while no statistically significant difference was noted between the L-DHI group and the TAC group (*P* > 0.05) (Figures 1(c) and 1(d)). We observed a significantly decreased LVAW (*P* < 0.05) in the H-DHI group compared with that in the TAC group (Figures 1(e) and 1(f)). Tissue Doppler was also performed to evaluate the cardiac diastolic function. We found that the Ea in the H-DHI group was improved compared with that in the TAC group (*P* < 0.05), whereas there was no statistically significant difference in the Ea/Aa (*P* > 0.05) (Figures 1(g) and 1(h)).

### 3.2. DHI Treatment Alleviated TAC-Induced CF

**Figure 2:**
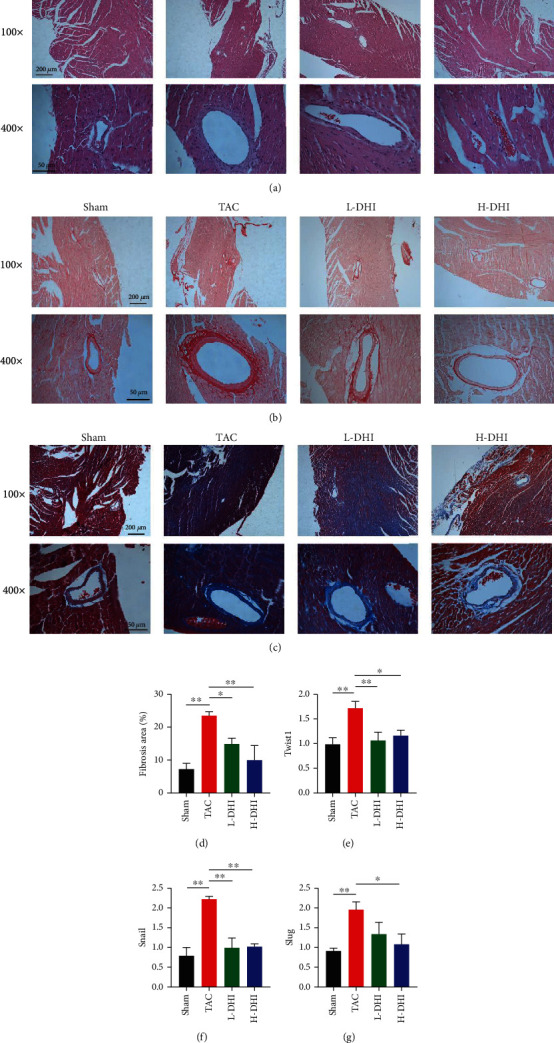
DHI treatment alleviated TAC-induced CF. (a) Typical H&E staining slides of hearts in various groups under 100x and 400x magnifications. (b) Typical Picrosirius red staining slides of hearts in various groups under 100x and 400x magnifications. (c) Typical Masson's trichrome staining slides of hearts in various groups under 100x and 400x magnifications. Blue represents the myocardial fibrotic area. Red represents the normal myocardium tissue. (d) The rate of the fibrotic area was measured on Masson's trichrome-stained slides and compared among groups on 28 days after TAC surgery (*n* = 3). (e) Expression of Twist1 mRNA in hearts was compared among groups (*n* = 3). (f) Expression of Snail mRNA in hearts was compared among groups (*n* = 3). (g) Expression of Slug mRNA in hearts was compared among groups (*n* = 3). ^∗^*P* < 0.05. ^∗∗^*P* < 0.01.

Since cardiac hypertrophy and remodeling were accompanied by the deposition of ECM, we evaluated cardiac fibrosis using H&E, Picrosirius red, and Masson's trichrome staining methods. The TAC surgery induced cardiac fibrosis in both the perivascular and interstitial areas, which was attenuated in the DHI-treated mice (Figures 2(a)–2(c)). The ratio of fibrotic area was significantly increased in the heart of the TAC group compared with that of the sham-operated group (*P* < 0.01), and that was declined in the H-DHI group (*P* < 0.05) (Figure 2(d)). The expressions of epithelial-mesenchymal transition-inducing transcription factors (EMT-TFs) (Twist1, Snail, and Slug) in the TAC group were noticeably elevated compared with those in the sham-operated group (*P* < 0.01). Treatment with H-DHI downregulated EMT-TF expression, as shown by decreased expressions of Twist1 (*P* < 0.05), Snail (*P* < 0.01), and Slug (*P* < 0.05) (Figures 2(e)–2(g)).

### 3.3. DNA Methylation Changes Induced by TAC

To identify DNA methylation changes induced by TAC, we performed methyl-seq with DNA samples obtained from TAC and sham-operated groups. The samples were subjected to the Agilent SureSelect Methyl-seq Target Enrichment System and then sequenced on an Illumina HiSeq X Ten System. This generated an average of ~93 million and 103 million reads for hearts of TAC and sham-operated mice, respectively, which uniquely aligned to the bisulfite-converted mouse reference genome (mm10). About 78% (TAC) and 77% (sham) of aligned reads remained after deduplication, which covered ~86% of targeted regions. The mean coverage of targeted regions was 72X (TAC) and 78X (sham). About 78% of targeted bases were observed to have coverage of at least 10X. An overview of these results is described in Table 3.

**Figure 3:**
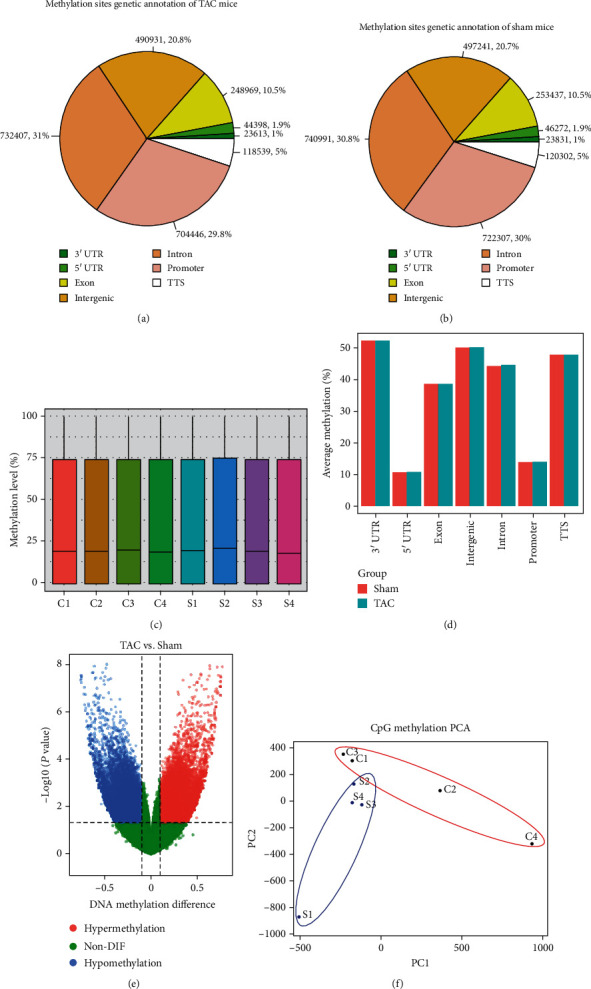
DNA methylation changes induced by TAC. (a) Methylation sites in genetic annotation of TAC mice. (b) Methylation sites in genetic annotation of sham-operated mice. (c) The total methylation levels of heart samples were compared between TAC (C1, C2, C3, and C4) and sham-operated (S1, S2, S3, and S4) groups. (d) The average methylation levels in landscape regions were compared between TAC and sham-operated groups (*n* = 4). (e) DNA methylation differences, including 19,618 hypermethylated genes and 22,988 hypomethylated genes, were detected between TAC and sham-operated groups. (f) PCA was performed to identify the clustering profiles of the methylated alterations induced by TAC. The red circle clustered samples from TAC mice (C1, C2, C3, and C4), and the blue circle clustered samples from sham-operated mice (S1, S2, S3, and S4).

We report here on our evaluation of DMSs in the CpG context. A total of ~2.3 million CpGs were commonly captured with a minimum coverage of 10X and good-quality base calls (<Q30). As illustrated in Figures 3(a) and 3(b), the majority of the DMSs were located in the promoter (~30%), the intron (~31%), and the distal intragenic regions (~21%). We compared the methylation levels of heart samples and found that global methylation was not significantly different between the TAC and sham-operated groups (Figure 3(c)). Average methylation levels were not remarkably different between the two groups within each region (Figure 3(d)). At the individual gene level, we noted a small subset of DMSs, including 19,618 hypermethylated DMSs and 22,988 hypomethylated DMSs (Figure 3(e)). We performed principal component analysis (PCA) to identify the clustering profiles of the methylated alterations induced by TAC. All the samples from the TAC group were clustered together and were slightly separated from the sham-operated group (Figure 3(f)). The above-mentioned results revealed that TAC induced the methylated changes of a small proportion of genes, rather than the overall proportion.

### 3.4. Methylated Regulation of Ras Signal Transduction Was Involved in the CF Development

**Figure 4:**
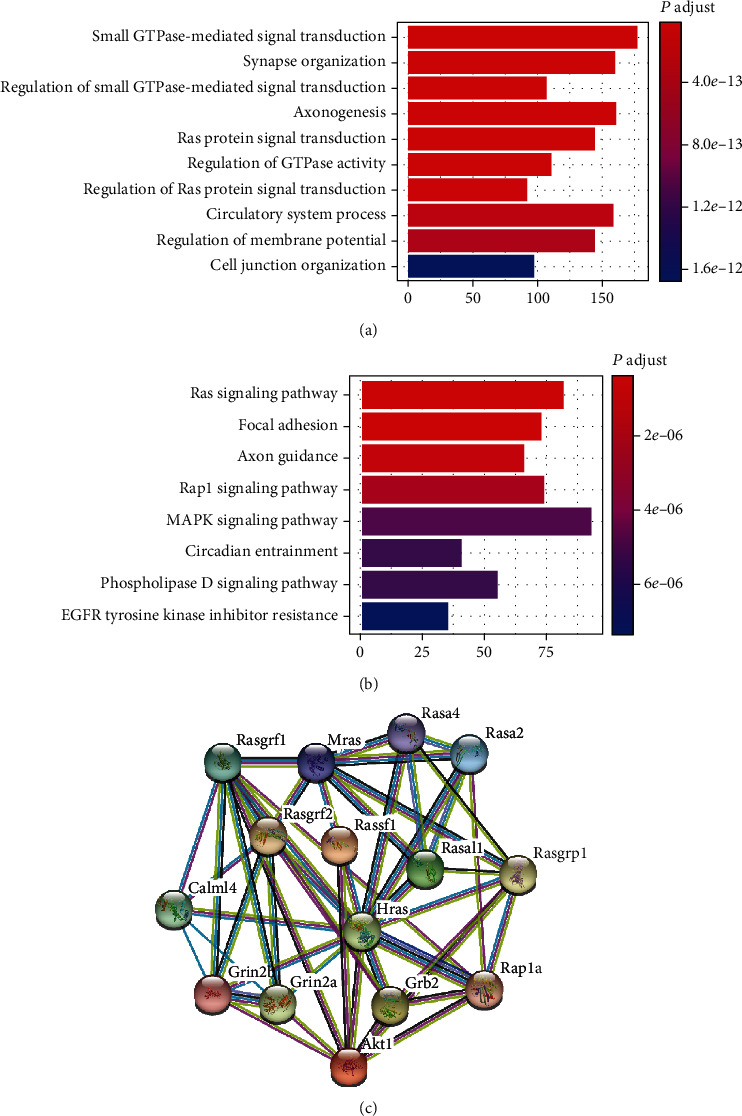
Methylated regulation of Ras signal transduction was involved in the CF development. (a) Results of GO enrichment analysis of DMSs. (b) Results of KEGG pathway analysis of DMSs. (c) Protein-protein interactions of Ras-related genes were observed in the STRING database.

To further explore the TAC-induced DNA methylated regulation of signal transduction pathways, GO enrichment and KEGG pathway analyses of DMSs were conducted. In the GO enrichment analysis, highly enriched clusters of “small GTPase-mediated signal transduction,” “regulation of small GTPase-mediated signal transduction,” “Ras protein signal transduction,” “regulation of GTPase activity,” and “regulation of Ras protein signal transduction” were found (Figure 4(a)). Furthermore, the KEGG pathway analysis indicated that Ras-related pathways, such as “focal adhesion,” “Rap1 signaling pathway,” and “MAPK signaling pathway,” were also enriched (Figure 4(b)). These results suggested that the methylated regulation of Ras signal transduction and focal adhesion-related genes were involved in the TAC-induced CF development. To specify the methylated genes, we analyzed the relationship among 20 genes with DMSs related to the Ras signaling pathway. PPIs were found between almost each gene, and a closed interaction between Rasal1, Rassf1, and Ras (Hras) was observed in the STRING database (Figure 4(c)).

### 3.5. DHI Treatment Downregulated DNMT3B Expression and Prevented the Hypermethylation of Rasal1 and Rassf1

**Figure 5:**
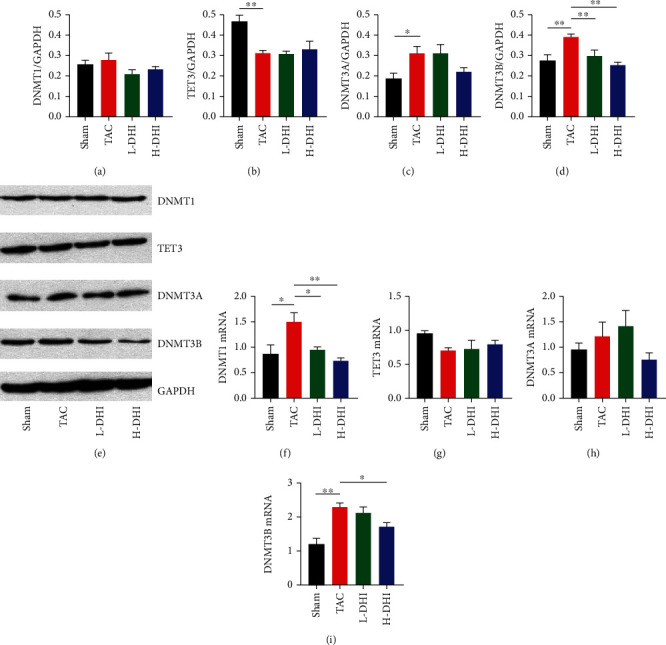
DHI treatment downregulates DNMT3B expression in CF hearts. (a) Quantification of DNMT1 protein level in CF hearts. (b) Quantification of TET3 protein level in CF hearts. (c) Quantification of DNMT3A protein level in CF hearts. (d) Quantification of DNMT3B protein level in CF hearts. (e) Representative Western blots of DNMT1, TET3, DNMT3A, DNMT3B, and GAPDH. (f) Relative mRNA level of Dnmt1 in CF hearts. (g) Relative mRNA level of Tet3 in CF hearts. (h) Relative mRNA level of Dnmt3a in CF hearts. (i) Relative mRNA level of Dnmt3b in CF hearts. ^∗^*P* < 0.05. ^∗∗^*P* < 0.01.

To investigate the mechanisms underlying the methylation of the Ras signaling pathway induced by TAC and the antifibrosis effect of DHI, we focused our investigation on the proteins and genes that mediated DNA methylation. In the CF hearts, we found a noticeable increase in the protein levels of DNMT3A (*P* < 0.05) and DNMT3B (*P* < 0.01) and a decrease in the TET3 (*P* < 0.01). After the DHI treatment, a significant downregulation of DNMT3B was observed in hearts from L-DHI and H-DHI groups (*P* < 0.01), while no significant changes in the levels of DNMT1, DNMT3A, and TET3 were noted (*P* > 0.05) (Figures 5(a)–5(e)). As shown in Figures 5(f)–5(i), the mRNA levels of Dnmt1 (*P* < 0.05) and Dnmt3b (*P* < 0.01) were elevated in CF hearts, and the H-DHI treatment significantly downregulated mRNA levels of Dnmt1 (*P* < 0.01) and Dnmt3b (*P* < 0.05).

**Figure 6:**
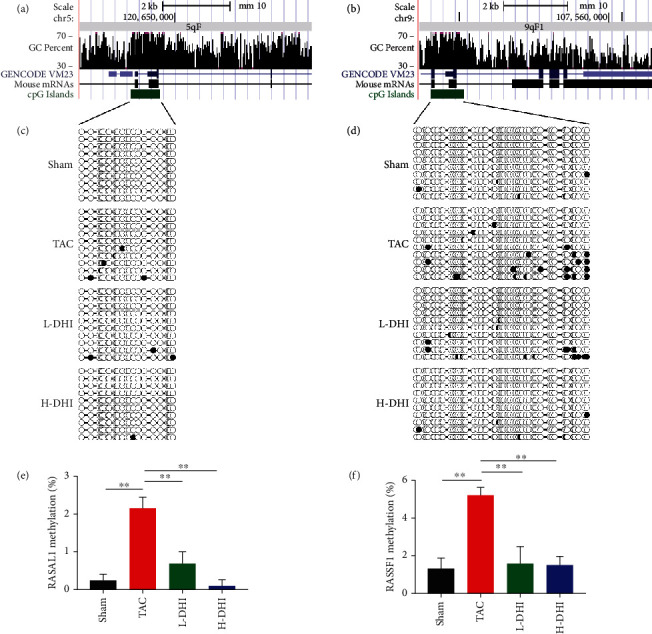
Bisulfite sequencing results of the Rasal1 and Rassf1 gene clusters. (a) Alignment results of bisulfite sequencing reads in the Rasal1 gene cluster are shown in the UCSC genome browser. (b) Alignment results of bisulfite sequencing in the Rassf1 gene cluster are illustrated in the UCSC genome browser. (c) The bisulfite PCR products corresponding to the region in the Rasal1 gene shown in (a) were analyzed using QUMA. (d) The bisulfite PCR products corresponding to the region in the Rassf1 gene shown in (b) were analyzed using QUMA. Each circle indicates a CpG site, and the methylated site is indicated in black. (e) QUMA analysis of the Rasal1 gene is shown as a bar graph. (f) QUMA analysis of the Rassf1 gene is displayed as a bar graph. Each bar represents the average methylation level of all CpG sites analyzed by each bisulfite product. Error bar is generated from 3 replicates. ^∗∗^*P* < 0.01.

Since the methylation regulating Ras signaling involved in the CF development and the hypermethylation of Rasal1 and Rassf1 genes after TAC were reported in the present and previous studies [3, 12], we attempted to consolidate the methylation levels of Rasal1 and Rassf1 genes and the effect of DHI by using bisulfite sequencing PCR. Alignment results of bisulfite sequencing reads in the Rasal1 and Rassf1 genes are displayed in Figure 6. The bisulfite sequencing PCR results compatible with the direction of changes were observed in the methyl-seq data, and the TAC-induced methylation affected the CpG site in both Rasal1 and Rassf1 genes. We found that the methylation of Rasal1 and Rassf1 in CF hearts was markedly downregulated by the DHI treatment (*P* < 0.01) (Figures 6(c)–6(f)).

### 3.6. DHI Treatment Upregulated Expressions of Rasal1 and Rassf1 Genes and Inhibited the Hyperactivity of Ras/ERK

**Figure 7:**
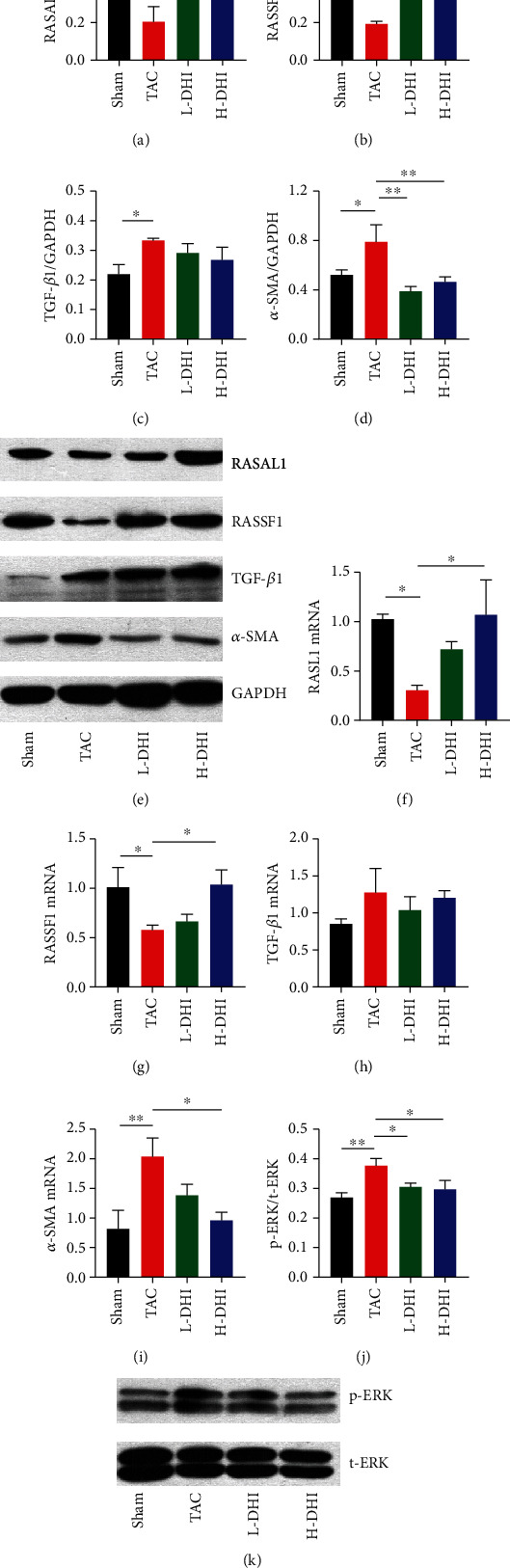
DHI treatment regulates expressions of Rasal1 and Rassf1 and reduced the hyperactivity of Ras/ERK in CF hearts. (a) Quantification of RASAL1 protein level in CF hearts. (b) Quantification of RASSF1 protein level in CF hearts. (c) Quantification of TGF-*β*1 protein level in CF hearts. (d) Quantification of *α*-SMA protein level in CF hearts. (e) Representative Western blots of RASAL1, RASSF1, TGF-*β*1, *α*-SMA, and GAPDH. (f) Relative mRNA level of Rasal1 in CF hearts. (g) Relative mRNA level of Rassf1 in CF hearts. (h) Relative mRNA level of TGF-*β*1 in CF hearts. (i) Relative mRNA level of Acta2 (*α*-SMA) in CF hearts. (j) Phosphorylated level of ERK in CF hearts. (k) Representative Western blots of phosphorylated and total ERK. ^∗^*P* < 0.05. ^∗∗^*P* < 0.01.

RASAL1 and RASSF1 bind to Ras in a GTP-dependent manner, converting active Ras-GTP to inactive Ras-GDP, and therefore function as inhibitors of Ras signaling [20, 21]. The activity of the Ras/ERK pathway was suggested to be important to maintain TGF*β*-induced fibrosis [22]. As illustrated in Figure 5(h), the expression of TGF-*β*1 in hearts was significantly increased after 28 days of TAC surgery (*P* < 0.05), and that was not affected by DHI treatment (*P* > 0.05). Consistent with the results of bisulfite sequencing, the results of Western blotting revealed that the protein expressions of Rasal1 and Rassf1 were markedly depressed in CF hearts (*P* < 0.05), and DHI treatment significantly upregulated protein expressions of Rasal1 (*P* < 0.05) and Rassf1 (*P* < 0.01) (Figures 7(a) and 7(b)). The results of RT-qPCR revealed similar findings in terms of the mRNA expressions of Rasal1 (*P* < 0.05) and Rassf1 (*P* < 0.01) (Figures 7(f) and 7(g)). To investigate the activity of the Ras/ERK pathway, we measured the phosphorylated level of ERK at Thy202/Tyr204 using Western blotting analysis. As shown in Figures 7(j) and 7(k), phosphorylation of ERK was significantly elevated in CF hearts (*P* < 0.01), and this ERK hyperactivity was inhibited by both L- and H-DHI treatments (*P* < 0.05). Besides, *α*-SMA is a marker for cardiac myofibroblasts in hypertrophic and fibrotic hearts [23]. As depicted in Figures 7(d) and 7(i), the mRNA (*P* < 0.01) and protein (*P* < 0.05) levels of *α*-SMA were noticeably increased in the fibrotic hearts compared with those in the sham hearts and were downregulated by H-DHI treatment (*P* < 0.05, *P* < 0.01).

## 4. Discussion

CF is characterized by net accumulation of ECM proteins in the cardiac interstitium and contributes to both systolic and diastolic dysfunction in several cardiac pathophysiologic conditions [24]. However, there is no therapy for cardiac fibrosis in general, which is largely due to the complex underlying basis of CF. DHI, a modern patented Chinese herbal medicine prepared from two cardiovascular medicinal herbs—*Salvia miltiorrhiza* (Dan Shen) and *Flos carthami* (Hong Hua, safflower), is widely used in China due to its safety and efficacy in treating cardiovascular and cerebrovascular diseases [25]. Both herbs are well known to improve blood circulation, eliminate blood stasis, and relieve menstrual pain [26]. According to the results of UPLC-TOF-MS, the main components in DHI are salvianolic acid A, salvianolic acid B, salvianolic acid D, kaempferide, coumalic acid, rosmarinic acid, and protocatechualdehyde (Supplementary Figure 1). Previous studies reported that DHI has inhibitory effects on CF and cardiac hypertrophy [10]. In rats with myocardial infarction, DHI treatment improved cardiac remodeling and preserves ventricular function by suppressing the expressions of TGF-*β*1 and fibrosis-related proteins (MMP-2 and MMP-9) [27]. Yang et al. pointed out that the *Salvia miltiorrhiza* and *Carthamus tinctorius* extract (SCE) prevents myocardial fibrosis and adverse remodeling after MI by suppressing histone methylation of the SMAD3 and its transcription in cardiac fibroblasts [10]. Additionally, a recent study showed that salvianolic acids, the main ingredients of DHI, have promising influences on some chronic fibrosis diseases, such as liver fibrosis and pulmonary fibrosis [28]. Consistent with the results of previous studies [10], we, in the present study, confirmed that the DHI treatment alleviated CF, improved cardiac function, and alleviated cardiac hypertrophy in TAC mice, with a superior effect at high doses.

A previous research has shown that DNA methylation regulates the expression of genes and is involved in the pathogenesis of CF [2]. In mammalian genomes, DNA methylation is a prevalent modification that decorates the majority of cytosines [29]. Gene silencing may occur due to methylation of DNA in the promoter region of genes [30]. To reveal the DNA methylation changes in CF hearts, we analyzed the methyl-seq data and then validated it by bisulfite sequencing. We found the methylated changes of a small proportion of genes involved in the development of CF. In our DMS analysis, the methylated regulation of Ras signal transduction and focal adhesion-related genes could be involved in the TAC-induced CF development, including Rasal1 and Rassf1.

Methylation of CpG islands is a prototypical epigenetic mechanism for controlling gene expression [31]. RASAL1 and RASSF1 function as inhibitors of Ras signaling and are involved in the pressure overload-induced CF. RASAL1 decreases the expression of p-ERK1/2, a downstream molecule in the RAS/RAF/MEK/ERK signaling pathway [20]. Xu et al. found that increased fibrosis was associated with significantly increased RASAL1 promoter methylation, decreased RASAL1 expression, and increased Ras-GTP activity [3]. In mice with pressure overload due to ascending aortic constriction, BMP7 significantly reduced RASAL1 promoter methylation, increased RASAL1 expression, and decreased cardiac fibrosis. Transcriptional suppression of RASAL1 through aberrant methylation of CpG islands contributes to the progression of cardiac fibrosis [3]. Upregulation of p-ERK1/2 was detected in activated cardiac fibroblasts with decreased RASSF1A expression, which is important for pathogenesis of CF and activation of fibroblasts [4]. With the aid of bisulfite sequencing, RT-qPCR, and Western blotting, we confirmed the TAC-induced changes in Rasal1 and Rassf1 genes at methylation, mRNA, and protein levels. In CF hearts, DHI treatment prevented the hypermethylation of Rasal1 and Rassf1, upregulated the expressions of Rasal1 and Rassf1, and inhibited the hyperactivity of Ras/ERK.

To investigate the mechanisms underlying the antifibrosis and the methylation regulatory effect of DHI, we investigated the enzymes mediating DNA methylation in mammals. In the present study, we observed an increase in expressions of the DNMT1 and DNMT3B and a decrease in the expression of TET3. Notably, treatment with DHI downregulated DNMT3B expression at both protein and mRNA levels in CF hearts. The changes in expressions of DNMT1, DNMT3B, and TET3 in CF hearts were previously reported. Watson et al. found that the hypoxia-induced profibrotic changes were associated with global DNA hypermethylation and increased expressions of DNMT1 and DNMT3B [32]. Xu et al. pointed out that among the three TET family enzymes (TET1, TET2, and TET3), only TET3-mediated hydroxymethylation causally contributes to reversal of endothelial-to-mesenchymal transition (EndMT) [3]. The inhibitory effect of salvianolic acid B, a main component of DHI, on DNA methylation in fibrotic disease through regulating DNMTs has been previously reported, while the underlying mechanism remains to be clarified [11]. Previous researches revealed that hypoxia and elevated cAMP are involved in the altered expression of these DNA methylation enzymes in CF [32]. Since the protective effect of DHI against hypoxia has been widely reviewed [25], the DHI-induced downregulation of DNMT3B in CF hearts could be mediated via hypoxia signaling pathways, while further investigation is essential.

Administration of the DNA methylation inhibitor could relieve the CF and hypertrophy and improve the cardiac function. In a spontaneously hypertensive rat (SHR) model, administration of DNMT inhibitors remarkably improved echocardiographic parameters associated with hypertrophy and diastolic dysfunction [33]. Myocardial collagen levels and myocyte size were reduced in 5-aza-treated SHR [34]. Type I collagen, type II collagen, and *α*-SMA were reduced in a human ventricular cardiac fibroblast cell line treated with 5-aza [34]. Several DNMT inhibitors have been discovered in the past years, while there is lack of a highly efficient, specific, and safe compound [6, 35]. On the contrary, the safety of DHI is tangible [25]. A study analyzed the drug use patterns of 84,697 patients who were first diagnosed with coronary heart disease in 17 hospitals and demonstrated that the most commonly used Chinese herbal medicine was DHI [34]. The adverse drug reaction (ADR) analysis of DHI was performed on 30,888 patients from 37 hospitals in 6 provinces across China. It was unveiled that the majority of the ADRs associated with DHI therapy were mild and moderately severe. The main treatment for DHI-associated ADRs is discontinuation of therapy [36].

Mechanisms that mediate aberrant methylation of select genes in the heart are still unknown. Studies in cancer or kidney fibrosis suggest that aberrant methylation is caused by pathological activity of DNA methyltransferases; what directs DNMTs to specific genes is yet entirely unknown [3]. In the present study, we reported that the perturbation of DNA methyltransferase presented in the development of CF which was alleviated by DHI. It was reported that the aberrant promoter methylation of Rasal1 and Rassf1 contributes to pressure overload-induced CF [3, 4]. Based on these studies, we found that TAC-induced methylation affected CpG sites in Rasal1 and Rasff1 genes and DHI treatment prevented the hypermethylation in these genes. Focusing on the methylation balancing effect of DHI in vivo, the direct relationship between DNMT3B and Ras-signal gene hypermethylation in vitro assays needs further exploration.

In summary, the present study proposed an intricate epigenetic mechanism, in which the delivery of DHI prevented CF. We found that epigenetic regulation of Rasal1 and Rassf1 was involved in the CF development. DHI treatment alleviated CF, prevented the hypermethylation of Rasal1 and Rassf1, and downregulated DNMT3B expression in CF hearts. The current research provided a novel treatment strategy for pressure overload-induced CF patients. However, focusing on the methylation balancing effect of DHI in vivo, we did not investigate the direct relationship between DNMT3B and Ras-signal gene hypermethylation in vitro assays, and further exploration is required to elucidate the benefits of DHI as a complementary therapy for diastolic heart failure and its methylation effect.

## Figures and Tables

**Table 1:** Primers used for transcript analysis by qRT-PCR.

Gene		Sequence
Gapdh	Forward	CAAGCTCATTTCCTGGTATGACA
	Reverse	TCTCTTGCTCAGTGTCCTTGCT

Dnmt3a	Forward	ACAGGTTTTCTCATGGGCACT
	Reverse	CCATGCAGCCATTTGAAAGTA

Acta2 (*α*-SMA)	Forward	TGGCTGTTCTTGCAGAAGACC
	Reverse	GTGCCAGCAAAGGTCAGAGAA

Rasal1	Forward	CGGACTCAGAAAAGCATTCAAT
	Reverse	GCTGCCAGATGAGAGTGGAATA

Twist1	Forward	GCCTGCAAAATCATAGTCAGTGA
	Reverse	TGCATTTAGACACCGGATCTATT

Snail	Forward	CAGCTGCTTCGAGCCATAGA
	Reverse	CCAGTAACCACCCTGCTGAG

Slug	Forward	CTGTATGGACATCGTCGGCA
	Reverse	ATGGGGGTCTGAAAGCTTGG

Tgf-b1	Forward	AGGGCTACCATGCCAACTTC
	Reverse	CCACGTAGTAGACGATGGGC

Rassf1	Forward	AGCGTGCCAACGCTCT
	Reverse	AACGGTAATGGCAGGTGAACT

Dnmt3b	Forward	GCCAGACCTTGGAAACCTCA
	Reverse	GCTGGCACCCTCTTCTTCAT

Dnmt1	Forward	GTCTTCCCCCACTCTCTTGC
	Reverse	ATCTTGCAGGTTGCAGACGA

Tet3	Forward	GGGCAGGCAGCGTAGC
	Reverse	ATGAGGTGAGCCAATGGGTG

**Table 2:** Primers used for DNA methylation analysis by bisulfite sequencing PCR.

Gene		Sequence
Rasal1	Forward	GAGATAATTTTTATGAAAGGTTTAAG
	Reverse	ATCCC(G/A)AAACAATACTCTCTCTAA

Rassf1	Forward	TTTTTGAAAGGGTTTATTTTTGTGT
	Reverse	CCCACAACTATAATACTACCTCCCTT

**Table 3:** Sequencing metrics obtained from the mouse methyl-seq platform. Values shown are the average of 3 independent samples from each group.

Sequencing metrics	TAC (*n* = 4)	Sham (*n* = 4)
Raw reads	130,818,980	145,491,597
Bad-quality reads	591,371	649,678
Bad-quality read ratio	0.5%	0.5%
Paired-end reads (PER)	129,642,360	144,198,984
Uniquely mapped paired-end reads (UMPER)	93,075,304	103,311,160
Mapping efficiency (UMPER/PER)	72%	72%
Duplicate read ratio (% of UMPER)	22%	23%
Duplicated UMPER	20,945,393	24,365,265
Target reads	62,107,357	67,942,565
Target read ratio	86%	86%
Deduplication reads	108,696,967	119,833,720
Capture ratio	57%	57%
Average read depth coverage (X)	72	78
Percent of target base covered at least 10X	78%	78%

## Data Availability

The data used to support the findings of this study are available from the corresponding author upon request.

## Supplementary Materials

Supplementary Materials Figure 1. Total ion chromatograms of DHI in positive (A) and negative ion mode (B) based on UPLC-TOF MS. P1: Protocatechualdehyde; P2: Salvianolic acid D; P3: Kaempferide; P4: Salvianolic acid A; P5: Coumalic acid; P6: Rosmarinic acid; P7: Salvianolic acid B.
