# Supplementary material for: Danhong Injection Alleviates Cardiac Fibrosis via Preventing the Hypermethylation of Rasal1 and Rassf1 in TAC Mice
Source: Oxid Med Cell Longev. 2020 Dec 29;2020:3158108. doi: 10.1155/2020/3158108 (PMC7787771; doi:10.1155/2020/3158108)
Supplement: Supplementary Materials — Figure 1. Total ion chromatograms of DHI in positive (A) and negative ion mode (B) based on UPLC-TOF MS. P1: Protocatechualdehyde; P2: Salvianolic acid D; P3: Kaempferide; P4: Salvianolic acid A; P5: Coumalic acid; P6: Rosmarinic acid; P7: Salvianolic acid B. [file 3158108.f1.docx]

## Supplementary Materials


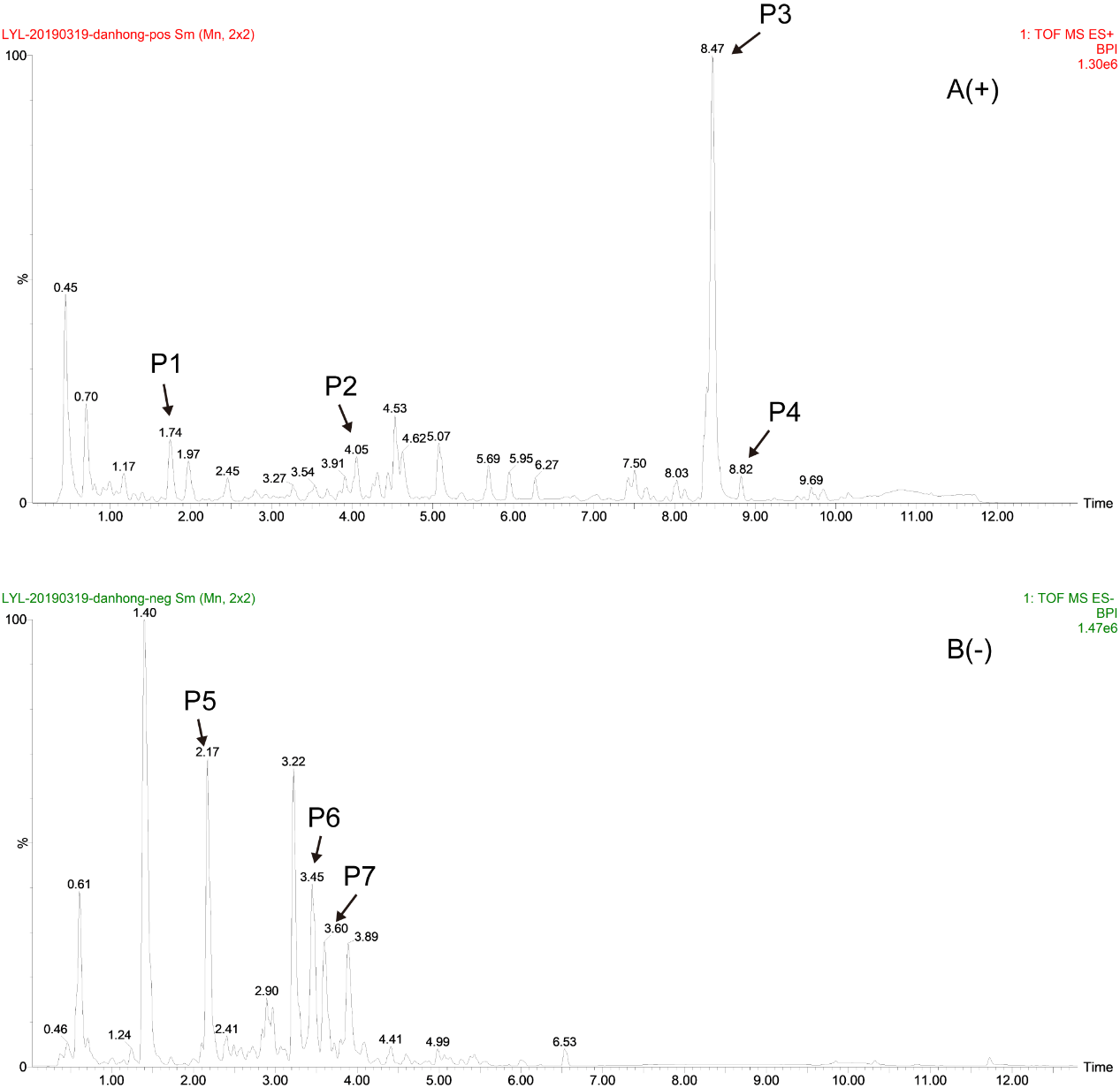


Figure 1. Total ion chromatograms of DHI in positive (A) and negative ion mode (B) based on UPLC-TOF MS. P1: Protocatechualdehyde; P2: Salvianolic acid D; P3: Kaempferide; P4: Salvianolic acid A; P5: Coumalic acid; P6: Rosmarinic acid; P7: Salvianolic acid B.
